# Level of engagement of recreational physical activity of urban villagers in Luohu, Shenzhen, China

**DOI:** 10.1371/journal.pone.0258085

**Published:** 2021-10-28

**Authors:** Lu Shi, Willie Leung, Qingming Zheng, Jie Wu

**Affiliations:** 1 Public Health, School of Social and Behavioral Health Science, College of Public Health and Human Sciences, Oregon State University, Corvallis, OR, United States of America; 2 Department of Health Sciences and Human Performance, College of Natural and Health Sciences, The University of Tampa, Tampa, FL, United States of America; 3 Shenzhen Luohu Disease Prevention and Control Center, Shenzhen, Guangdong, China; La Inmaculada Teacher Training Centre (University of Granada), SPAIN

## Abstract

Physical activity is important for health. However, there is a lack of literature related to the physical activity levels of adults living in urban villagers, which is a vulnerable population in China. The aim of this study is to compare the physical activity and sedentary behavior engagements between urban villagers and non-urban villagers using the 2019 Luohu Shenzhen, China Community Diagnosis Questionnaire. A total of 1205 adults living in urban villages and non-urban villages were included in the analysis. Unadjusted and multiple multivariate logistic regression were conducted for the dependent variable of engagement in recreational physical activity, frequency of recreational physical activity per week, and hours spent in sedentary behaviors per day. Descriptive analysis was conducted to identify the reasons for not engaging in physical activity among urban villagers and non-urban villagers. Across the included sample, 29.05% were urban villagers and 70.95% were non-urban villagers. The results suggested that urban villagers are more likely to engage in physical activity than non-urban villager (OR = 1.90, 95% CI [1.40, 2.59], p < 0.001). However, it was also found that urban village status had no significant association for frequency in engaging in physical activity and average hours spent in sedentary behaviors. Both urban villagers and non-urban villages indicated that lack of time, lack of safe and appropriate environment, and working in labor intensive occupations as some of the reasons for not engaging in physical activity. There is a need for tailed interventions and policies for promoting physical activity among urban villagers and non-urban villagers. Additional studies are needed to further our understanding of the physical activity behaviors among urban villagers in China.

## Introduction

### Benefits of physical activity

The benefit of engagement of physical activity is well documented [1, 2]. The numerous benefits included weight management, lower blood cholesterol levels and blood pressure, strengthening bones, muscles, and joint, and reducing risk of cardiovascular disease and certain types of cancers [3]. In addition to physical health-related benefits, engagement in physical activity could lead to benefits of social and mental benefits. Regular engagement in physical activity is associated with reduced stress, improved mental health, emotional regulation, lowered depression, increased social functioning, and increased sense of community [4]. Further, engagement of regular physical activity is related to reduce the risk of developing disabilities and maintenance of functional independences [5, 6].

Currently, physical inactivity is the fourth leading cause of mortality, according to the World Health Organization (WHO) [7]. WHO’s physical activity guidelines are 150 minutes of moderate physical activity or 75 minutes of vigorous physical activity per week or an equivalent combination of moderate- and vigorous-intensity activity for adults [7]. Individuals can perform various activities, such as leisure time physical activity, active transportation, and occupational activities to accumulate the minutes required to meet the guidelines. These guidelines apply to all individuals regardless of gender, race, ethnicity, or income levels.

### Physical activity levels of Chinese people

Past literature had examined the physical activity levels individuals living in China [8, 9]. Using the data from the 2012 to 2015 China Hypertension Survey (CHS), it was found that 28.1% of Chinese adults were overweight and 5.2% were obese [10]. The results also found that regionals different of the prevalence of overweight and obesity different between Northern and Southern China with adults from Northern China more likely to be obese and overweight. According to a report published in the official Report on Cardiovascular Diseases in China 2017, 290 millions of Chinese adults are suffering from cardiovascular disease [11]. It was also found that China is facing a fast growing cardiovascular disease epidemic with a widening rural-urban disparities [12].

Similar physical activity trends found in Western countries were observed among Chinese adults as well. Trends such as male are more likely to engage in physical activity than female and older adults are less physical active than younger adults were found among individuals living in China [8, 9]. It was found that 66.3% of adults between the ages of 35 to 74 years were physically active according to the data from the International Collaborative Study of Cardiovascular Disease in Asia from 2000–2001 [9]. Using accelerometers to measure physical activity, it was found that Chinese adults in Shanghai spent 317 minutes per day in physical activity, while spent 509 minutes per day in sedentary behaviors [13]. It was reported that Chinese adults are more likely to report engaging in work-related or occupational physical activity (63.3%) than leisure time physical or recreational physical activity (24.5%) [9]. There were disparities between urban and rural residents with more rural residents (78.1%) spending time in physical activity than urban residents (21.8%) [9]. In addition to regional different, it was found that socioeconomic status (SES) impact physical activity levels among Chinese adults [14]. Using a community-based survey with 3567 adults living in Jiaxing, China, Chen et al. found that adults with lower SES are more likely to engage in household physical activity, adults with middle SES engages in higher intensity of occupational physical activity, and adults with higher SES levels were more likely to exercise but spent longer time in sedentary behaviors [14].

The physical activity of subpopulation of Chinese adults had been well examined, especially for adults with different living area (rural vs. urban) and SES [14, 15]. However, there is a lack of literature examining the physical activity levels of urban villagers. Urban villagers refer to the individuals living in urban village. Urban village or *chengzhongcun* are typically low quality and high density with many closely packed apartment blocks of between 2 and 8 floors [16]. Urban villages are transitional neighborhoods typically found in urban areas or cities with rapid economic growth [16, 17]. Urban villages can be described as narrow roads, face-to-face buildings, a thin strip of sky, and inner streets packed with shops, grocery stores and service outlets [16]. Many of these urban villages are associated with unsuitable land use, poor housing construction, severe infrastructure deficiencies, intensified social disorder, and deteriorated urban environment [18]. In addition, urban villages often have poor sanitary condition, where pipelines and drainage systems are poorly constructed and water flows over the ground along with garage [17]. Many urban villagers are individuals with low SES status due to financial situation. These urban villagers could include rural-to-urban migrants workers with limited skillsets and educations or individuals who recently graduated from colleges and universities. They are attracted to urban villages due to the cheap housing accommodation. Overall, these urban villagers aggregate in urban village in large cities, such as Guangzhou and Beijing with limited infrastructure and poor living environments due to affordable living accommodations.

Due to the unique living situations of urban villages and limited healthcare resources [19], urban villagers’ physical activity need to be better examined [20]. Knowing physical activity-related information of urban villagers could better design and develop interventions targeting the needs of urban villagers in the community. Regular engagement in physical activity is associated with better health-related outcomes [21], considering urban villagers is more at risk for poor health outcomes due to poor living situation [22, 23]. Previous studies had examined the physical activity levels of youths and adolescents living in urban village [24, 25]. Therefore, to better understand the physical activity levels of adult urban villagers, the purpose of this study is to compare the physical activity and sedentary behaviors engagements between urban villagers and non-urban villagers using the 2019 Luohu Shenzhen, China Community Diagnosis Questionnaire.

## Materials and methods

### Design and sample

This study is secondary data analysis using data from the 2019 Luohu Shenzhen, China Community Diagnosis Questionnaire. The questionnaire is part of a community health diagnosis program funded by the Center for Disease Control and Prevention of Shenzhen. Due to the unique status of Shenzhen as the Special Economic Zones (SEZ), it attracted various Chinese citizens with different background to settle in the areas. This allows assessments of health-related behaviors on various groups of Chinese citizens (e.g., household registration status, migrants status, employments status, income levels, etc.) within the same survey and living within the same area. The goal of the survey is to grasp the main health problems existing in the residents of Luohu District, determine the causes of community health problems, and determine the priority needs of the public health services and factors affecting residents’ health. The survey also served as an evaluation of Shenzhen residents satisfaction on the various healthcare institutes available to them, such as community health centers. The survey consisted of seven parts: 1) family demographics, 2) family medical history, 3) adults healthcare needs and access to healthcare, 4) health and quality of life of adults over the ages of 60 years old, 5) health, healthcare and reproductive healthcare needs of married women under the ages of 50 years old, 6) healthcare needs and health of children, and 7) examination of blood pressure, height, weight, hip length, and waist length. Data collection of the survey was approved by the IRB at Shenzhen Luohu Disease Prevention and Control Center. Analysis of the survey data was approved by the IRB at Oregon State University.

**Fig 1:**
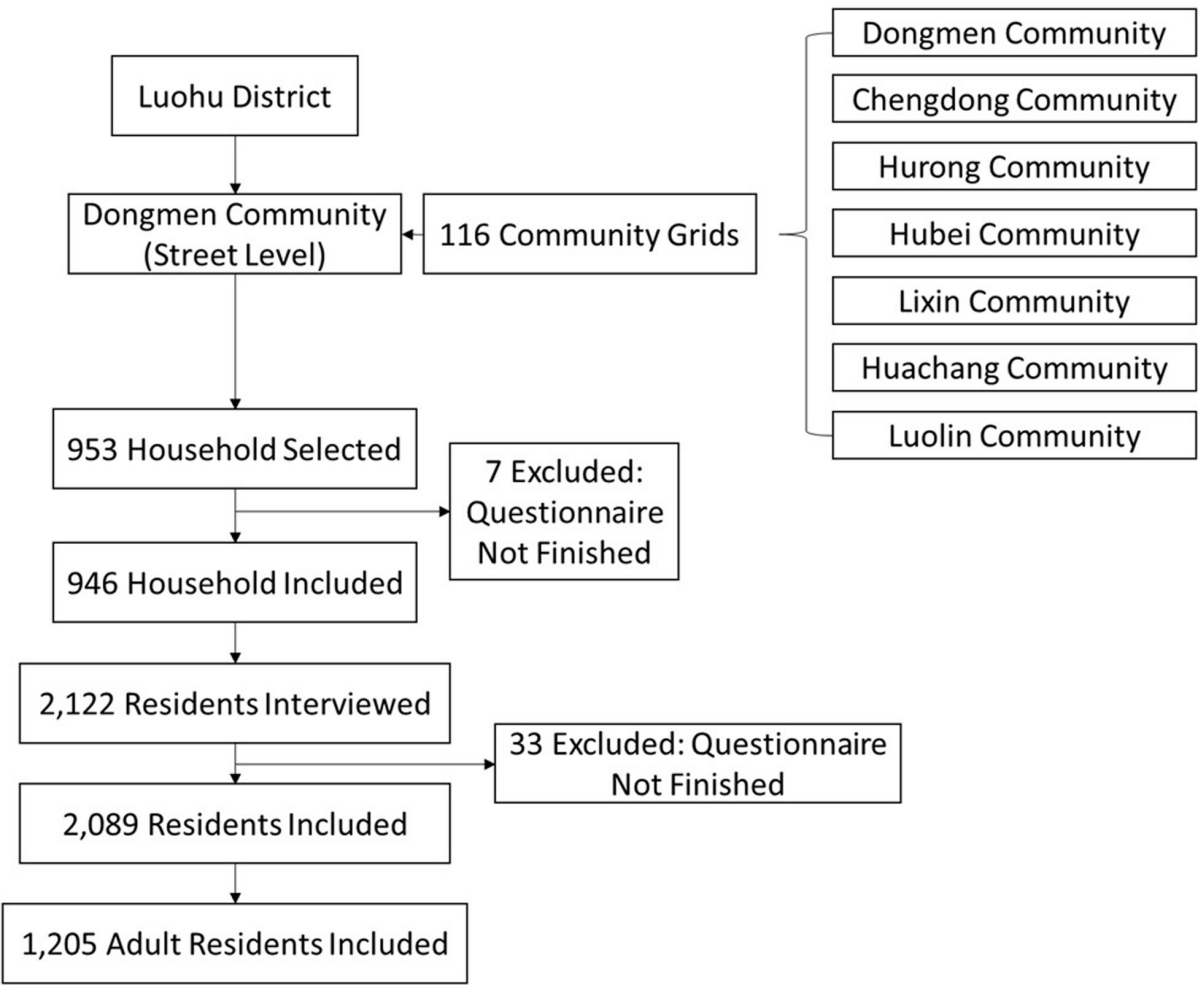
Participants recruitment process.

Participants of the survey were selected by multiple stages of random selection. First, seven communities were randomly selected in Dongmen community, Luohu district, Shenzhen, Luohu as seen in Fig 1. Then 116 community grids were randomly selected from the seven selected communities in Dongmen community, Luohu district. Lastly, family household, serving as survey unit, were randomly selected for interview based on the size of the community. All members of the household participated in the survey. Further, only individuals living in Shenzhen for at least six months prior to the interview were included in the survey. The number of household participants in the survey is based on the size of the community. 200 households were randomly selected if the community sample size have more than two million individuals, 150 households for community sample size between one to two million, 100 households for community sample size between half of a million to one million, and 50 households for community less than half of a million. The random selection of communities was to identify individuals living in the various type of communities within the Shenzhen area. All data were collected between January and September of 2019. All data were collected through face-to-face interview. A total of 2122 participants were interviewed for the survey. However, only 2089 participants completed the survey with valid data. 1205 adults were included in the analysis.

Across the sample, 54.52% of the participants were female and 45.48% of the participants were male. The average age of the participants were 38.8 years old. The average BMI were 22.88 kg/cm^2^ with the hip-to-waist ratio of .89. A majority of the participants were employed (83.24%). Interestingly, 32.20% of the participants have no formal education and completed primary education, which made up almost of one third of the sample. 28.54% of the participants completed middle school, 26.97% completed high school, and 12.28% completed professional school, college, and university. 76.02% of the participants were married or partnered and 23.98% were singled or not partnered. The sample consisted of more participants with non-Shenzhen *hukou* (62.41%). Across the sample, there were more participants without diagnosis of hypertension and diabetes. Only 6.56% and 1.91% of the participants reported having hypertension and diabetes, respectively. 76.43% of the participants reported they did not smoke.

### Measures

The independent variable of the analysis is the living status of the participants. The variable is based on the location of the community grids participants resides in. Shenzhen used the community grid system to identify local community [26]. Urban villages are typically located within one grid. Therefore, community grids serve as an indicator for urban villages. Participants were classified as urban villagers if they live in an urban village and all other participants were classified as non-urban villagers.

Physical activity and sedentary behavior-related variables from the survey component about health and quality of life of adults from 18 to 59 years old were selected for this analysis. A total of four variables were determined to be related to physical activity from the survey. The variables were engagement in recreational physical activity, frequency of recreational physical activity per week, hours spend in sedentary behaviors per day, and reasons for not engaging in physical activity. All variables were categorical variables. Engagement in recreational physical activity was based on the question of “Within the past six months, what types of recreational physical activity did you participated in?”. The respond options included: 1) did not participate in any activities, 2) machine equipment physical activity, 3) aerobic activity or aerobic dances, 4) swimming, 5) ambulatory activity (e.g., brisk walking, jogging, running, hiking), 6) ball-related sports (e.g. basketball, baseball, soccer, etc.), 7) sports or fitness competition, 8) martial arts, or 9) other. Participants were considered not to be engaged in physical activity when they responded with did not participate in any activities, else participants were classified as engaged in physical activity. Recreational physical activity is defined as physical activity that is done at leisure time. The variable of frequency of recreational physical activity were based on the question of “Within the past six months, how often do you exercise per week?” with the respond options of 1) 6 or more times per week, 2) 3 to 5 times per week, 3) 1 to 2 times per week, and 4) lesser than 1 time. The variable of hours spend in sedentary behaviors was based on the respond to the question of “In the past month, what is the average accumulated hours spend in sedentary activities (e.g., studying, working, watching TV, using computer, etc.)?”. The respond options included 1) lesser than 2 hours per day, 2) 2 to 4 hours per day, 3) 4 to 8 hours per day, 4) 8 to 12 hours per day, and 5) more than 12 hours per day. Reasons for not engaging in physical activity were only for participants who responded that they engaged in physical activity within the past six months. The survey item aims to identify how prevent them from engaging in physical activity throughout their routine. Participants were asked the reasons when they were unable to engage in physical activity weekly. Participants were able to select multiple options of 1) no recreational physical activity is needed due to labor intensive occupations, 2) no time to engage in physical activity, 3) there were no appropriate places and/or environments for physical activity, 4) I feel healthy, I do not need physical activity, 5) do not want to engage in physical activity, 6) feeling ill, unable to participate in physical activity, and 7) other reasons.

The covariates included in the analysis were gender, age, employment status, education, marital status, household registration, body mass index (BMI), diagnosis of hypertension, diagnosis of diabetes, and smoking status. Gender was a binary variable consisted of male and female. Age was a continuous variable between 18 to 59 years old. Employment status was a binary variable of being employed or unemployed. Education was a categorical variable including professional college and university, high school, middle school, and primary school or no formal education. Marital status was a binary variable of either being married or single. Household registration or *Hukou* were based on participants self-reporting their registration of either Shenzhen *Hukou* or non-Shenzhen *Hukou*. BMI is a continuous variable between 15.02 to 36.11 kg/cm^2^, which was calculated based on the participants’ height and weight by the survey. Hip-to-waist was calculated based on the hip and waist of the participants by the survey. Diagnosis of hypertension, diagnosis of diabetes, and smoking status were all binary variables with yes and no. These covariates were selected due to their relationship with physical activity engagement.

### Data analyses

Descriptive analysis was conducted for the independent variables, dependent variables, and the covariates. To determine the physical activity engagement between urban villagers and non-urban villagers, unadjusted and multiple multivariate logistic regression were conducted for the dependent variable of engagement in recreational physical activity, frequency of recreational physical activity per week, hours spend in sedentary behaviors per day, and reasons for not engaging in physical activity. All analyses were conducted using STATA version 16 (StataCorp LLC., College Station, TX, USA). The alpha levels were set at .05. The study protocol was approved by the Oregon State University (IRB: IRB-2020-0509).

## Results

Across the sample, 29.05% (n = 350) of the participants were urban villagers and 70.95% (n = 855) were non-urban villagers. Pearson’s chi square test found significant different between education levels, marital status, and household registration status between the urban villagers and non-urban villagers. There were more non-urban villagers with completed middle school, high school, and professional school, college, and university (χ^2^ = 99.46, *p* < 0.001). There were more non-urban villagers who were either married or partnered than urban villagers (χ^2^ = 3.77, *p* = 0.05). Regrading to household registration or *hukou*, there were higher proportion of non-urban villagers with Shenzhen *hukou* and higher proportion of urban villagers with non-Shenzhen *hukou* (χ^2^ = 180.60, *p* < 0.001). Also, there were significant different in age found between the two groups with non-urban villagers had a higher average age. Non-significant differences were found between urban villagers and non-urban villagers among other covariates (e.g., gender, employment, diagnosis of hypertension, diagnosis of diabetes, smoking status, BMI, and hip-to-waist ratio).

### Engagement in recreational physical activity

From the total sample size (n = 1205), 63.73% (n = 768) of participants reported not engage in any recreational physical activity while 36.27% (n = 474) reported engaged in recreational physical activity. A significant difference in proportion of engaging in recreational physical activity were found between urban and non-urban villagers (χ^2^ = 60.79, *p* < 0.001) with higher proportion of urban villagers (53.14%) reported engaging in recreational physical activity than non-urban villagers (29.36%) as shown in Table 1. The unadjusted logistic regression found that urban villagers were 2.73 (95% CI [2.11, 3.53], *p* < 0.001) times the odds of non-urban villagers in engaging in recreational physical activity as shown in Table 2. The results of the multivariate logistic regression found that Urban Villagers were 1.90 (95% CI [1.40, 2.57], *p* < 0.001) times the odds of non-urban villagers in engaging in recreational physical activity after controlling for covariates. The analysis also found the education levels, household registration, and BMI are significant factors contributing to the results of the odds ratios between urban villagers and non-urban villagers in engaging in recreational physical activity.

**Table 1:** Characteristics of urban villagers and non-urban villagers engaging in recreation physical activity.

	Urban Villagers		Non-Urban Villagers		Total			
	n	Mean/Proportion	n	Mean/Proportion	n	Mean/Proportion	χ^2^/ t	*P*
**Engagement in recreational physical activity, %**								
Yes	186	53.14	251	29.36	437	36.27	60.79	<0.001*
No	164	46.86	604	70.64	768	63.73		
**Frequency of recreational physical activity per week, %**								
> 6 times	35	21.34	154	25.45	189	21.75	1.19	0.76
3–5 times	57	34.76	201	33.22	358	41.20		
1–2 times	62	37.80	216	35.70	278	31.99		
< 1 time	10	6.10	34	5.62	44	5.06		
**Average hours spend in sedentary behaviors per day, %**								
> 12 hours	15	4.29	43	5.03	58	4.81	5.65	0.23
9–12 hours	46	13.14	113	13.22	159	13.20		
5–8 hours	99	28.29	221	25.85	320	26.56		
2–4 hours	86	24.57	261	30.53	347	28.80		
< 2 hours	104	29.71	217	25.38	321	26.64		
**Gender, %**								
Female	183	52.29	474	55.44	657	54.52	1.00	0.32
Male	167	47.71	381	44.56	548	45.48		
**Age, years**	350	37.75	855	39.24	1205	38.8	2.21	0.03*
**Employment status, %**								
Yes	302	86.29	701	81.99	1003	83.24	3.29	0.07
No	48	13.71	154	18.01	202	16.76		
**Education levels, %**								
College & university	46	13.14	342	40.00	148	12.28	99.46	<0.001*
High school	108	30.86	236	27.60	325	26.97		
Middle school	122	34.86	203	23.74	344	28.55		
Primary school & none	74	21.14	74	8.65	388	32.20		
**Marital Status**								
Married/partnered	253	72.29	663	77.54	916	76.02	3.77	0.05
Single	97	27.71	192	22.46	286	23.98		
**Household registration (*hukou*), %**								
Shenzhen *hukou*	29	8.29	424	49.59	453	37.59	180.60	0 < .001*
Non-Shenzhen *hukou*	321	91.71	431	50.41	752	62.41		
**Body Mass Index, kg/m** ^**2**^	350	22.99	855	22.83	1205	22.87	-0.70	0.48
**Hip-to-waist ratio, %**	350	89	855	89	1205	89	0.41	0.68
**Hypertension, %**								
Yes	19	5.43	60	7.02	79	6.56	1.02	0.31
No	331	94.57	795	92.98	1126	93.44		
**Diabetes, %**								
Yes	8	2.29	15	1.75	23	1.91	0.37	0.54
No	342	97.71	840	98.25	1182	98.09		
**Smoking status, %**								
Yes	94	26.86	190	22.22	284	81.66	2.96	0.09
No	256	73.14	665	77.78	921	18.34		

*Note*. n, sample size; *χ*^2^, chi-square statistic comparing between Urban Villagers and non-Urban Villagers for categorical variables; t, t-statistic comparing between Urban Villagers and non-Urban Villagers for continuous variables, *p*, p-value associated with the statistic comparison test;

*, *p* < 0.05.

**Table 2:** Odd ratios of urban villagers and non-urban villagers in engaging in recreational physical activity.

	Engagement in recreational physical activity			
	Unadjusted Model^b^		Adjusted Model^c^	
	OR	95% CI	OR	95% CI
Urban villagers	2.73*	2.11, 3.53	1.90*	1.40, 2.57
Non-urban villagers	1 (ref.)		1 (ref.)	

Abbreviations: OR, odds ratio; CI, confidence interval.

^a^Boldfaced numerals indicate p-value <0.05.

^b^Odd ratio from logistic regression model were computed for the outcome variable of engagement in recreational physical activity (yes/no) with the exposure variable of living situation (urban village/non-urban village).

^c^Odd ratio from multivariable logistic regression model were computed for the outcome variable of engagement in recreational physical activity (yes/no) with the exposure variable of living situation (urban village/non-urban village) adjusted for gender (male/female), age (continuous), employment status (yes/no), education levels (college & university, high school, middle school, primary school & none), marital status (married & partnered/single), household registration (*hukou*) (Shenzhen/non-Shenzhen), BMI (continuous), hip-to-waist ratio (continuous), hypertension (yes/no), diabetes (yes/no), and smoking status (yes/no).

^d^ Detail adjusted model outcome were showed in S1 Table.

### Frequency of recreational physical activity per week

21.34% of urban villagers reported engaging in recreational physical activity more than six times per week, in compared to 25.45% of non-urban villagers reported the same frequency. 34.76% of urban villagers and 33.72% of non- urban villagers reported engaging in recreational physical activity 3 to 5 time per week, 37.80% of urban villagers and 35.70% of non- urban villagers reported engaging recreational physical activity 1 to 2 times per week. And 6.10% of urban villagers and 5.62% of non-urban villagers reported engaged in lesser than recreational physical activity per week. No significant different was found between the two groups regarding the frequency of engaging recreational in physical activity per week (χ^2^ = 1.19, *p* = 0.76). The odds ratio of the unadjusted logistic regression for each level of the frequency of engaging in recreational physical activity per week with references of less than 1 time per week were 0.98 (95% CI [0.46, 2.09], *p* = 0.95) for 1 to 2 time per week, 0.96 (95% CI [0.45, 2.07], *p* = 0.93) for 3 to 5 times per week, and 0.77 (95% CI [0.35, 1.71], *p* = 0.95) for more than six times per week for urban villagers in engaging in recreational physical activity compared to non-urban villagers. The results of the multivariate logistic regress found that urban villagers status is not a significant factor in estimating the odds ratio of frequency in engaging recreational physical activity per week with the reference groups of lesser than 1 time per week as shown in Tables 3 and 4.

**Table 3:** Odd ratios of frequency of engaging in recreational physical activity per week between urban villagers and non-urban villagers.

	Unadjusted odd ratios^b^						Adjusted odd ratios^c^					
	1–2 times vs. < 1 time (ref.)		3–5 times vs. < 1 time (ref.)		> 6 times vs. < 1 time (ref.)		1–2 times vs. < 1 time (ref.)		3–5 times vs. < 1 time (ref.)		> 6 times vs. < 1 time (ref.)	
	OR	95% CI	OR	95% CI	OR	95% CI	AOR	95% CI	AOR	95% CI	AOR	95% CI
Urban villagers	0.98	0.46, 2.09	0.96	0.45, 2.07	0.77	0.35, 1.71	1.07	.44, 2.64	0.98	.39, 2.43	0.83	.32, 2.15
Non-urban villagers	1 (ref.)		1 (ref.)		1 (ref.)		1 (ref.)		1 (ref.)		1 (ref.)	

Abbreviations: OR, odds ratio; CI, confidence interval.

^a^Boldfaced numerals indicate p-value <0.05.

^b^Odd ratio from logistic regression model were computed for the outcome variable of engagement in recreational physical activity (yes/no) with the exposure variable of living situation (urban village/non-urban village).

^c^Odd ratio from multivariable logistic regression model were computed for the outcome variable of engagement in recreational physical activity (yes/no) with the exposure variable of living situation (urban village/non-urban village) adjusted for gender (male/female), age (continuous), employment status (yes/no), education levels (college & university, high school, middle school, primary school & none), marital status (married & partnered/single), household registration (*hukou*) (Shenzhen/non-Shenzhen), BMI (continuous), hip-to-waist ratio (continuous), hypertension (yes/no), diabetes (yes/no), and smoking status (yes/no).

^d^ Detail adjusted model outcome were showed in S2 Table.

**Table 4:** Odd ratios of average hours spend in sedentary behaviors per day between urban villagers and non-urban villagers.

	2–4 hours vs. < 2 hours		5–8 hours vs. < 2 hours		9–12 hours vs. < 2 hours		>12 hours vs. < 2 hours		2–4 hours vs. < 2 hours		5–8 hours vs. < 2 hours		9–12 hours vs. < 2 hours		>12 hours vs. < 2 hours	
	OR	95% CI	OR	95% CI	OR	95% CI	OR	95% CI	OR	95% CI	OR	95% CI	OR	95% CI	OR	95% CI
Urban villagers	0.69*	.49, .96	0.93	.67, 1.30	0.85	.56, 1.29	0.73	.39, 1.37	0.85	.58, 1.25	1.18	.79, 1.75	1.43	.86, 2.38	0.06	0, 2.07
Non-urban villagers	1 (ref.)		1 (ref.)		1 (ref.)		1 (ref.)		1 (ref.)		1 (ref.)		1 (ref.)		1 (ref.)	

Abbreviations: OR, odds ratio; CI, confidence interval.

^a^Boldfaced numerals indicate p-value <0.05.

^b^Odd ratio from logistic regression model were computed for the outcome variable of engagement in recreational physical activity (yes/no) with the exposure variable of living situation (urban village/non-urban village).

^c^Odd ratio from multivariable logistic regression model were computed for the outcome variable of engagement in recreational physical activity (yes/no) with the exposure variable of living situation (urban village/non-urban village) adjusted for gender (male/female), age (continuous), employment status (yes/no), education levels (college & university, high school, middle school, primary school & none), marital status (married & partnered/single), household registration (*hukou*) (Shenzhen/non-Shenzhen), BMI (continuous), hip-to-waist ratio (continuous), hypertension (yes/no), diabetes (yes/no), and smoking status (yes/no).

^d^ Detail adjusted model outcome were showed in S3 Table.

### Average hours spend in sedentary behaviors per day

4.29% of urban villagers and 5.03% non-urban villagers reported spending more than 12 hours per day in sedentary, which made up the smallest proportion of the participants in their respective group. 13.14% of urban villagers and 13.22% of non-urban villagers reported spending 8 to 12 hours per day in sedentary behaviors. 28.29% of urban villagers and 25.85% of non-urban villagers reported spending 4 to 8 hours per day on sedentary behaviors, while 24.57% and 30.53% of urban villagers and non-urban villagers spend 2 to 4 hours per day on sedentary behaviors. For lowest amount of time spend in sedentary behaviors, 29.71% of urban villagers and 25.36% of non-urban villagers reported spending lesser than 2 hours on it. Non-significant different was found between the two groups regarded to the self-reported hours spend in sedentary hours (χ^2^ = 5.65, *p* = 0.23). From the unadjusted logistic regression with the reference group of spending less than 2 hours per day in sedentary behaviors and urban villagers, the odd ratios were 0.69 (95% CI [0.49, 0.96], *p* = 0.03) for 2 to 4 hours, 0.93 (95% CI [0.67, 1.30], *p* = .69) for 4 to 8 hours, and 0.85 (95% CI [0.56, 1.29], *p* = 0.44) for 8 to 12 hours. The results of the multivariate logistic regression found that urban villagers status is not a significant factor in estimating the hours spend in sedentary behaviors per day with the reference groups of lesser than 2 hours per day as shown in Table 4. However, across all levels of hours spend in sedentary behaviors, completing professional school, college, and university had a higher odd of spending more time in sedentary behaviors.

### Reasons for not engagement in recreational physical activity

**Fig 2:**
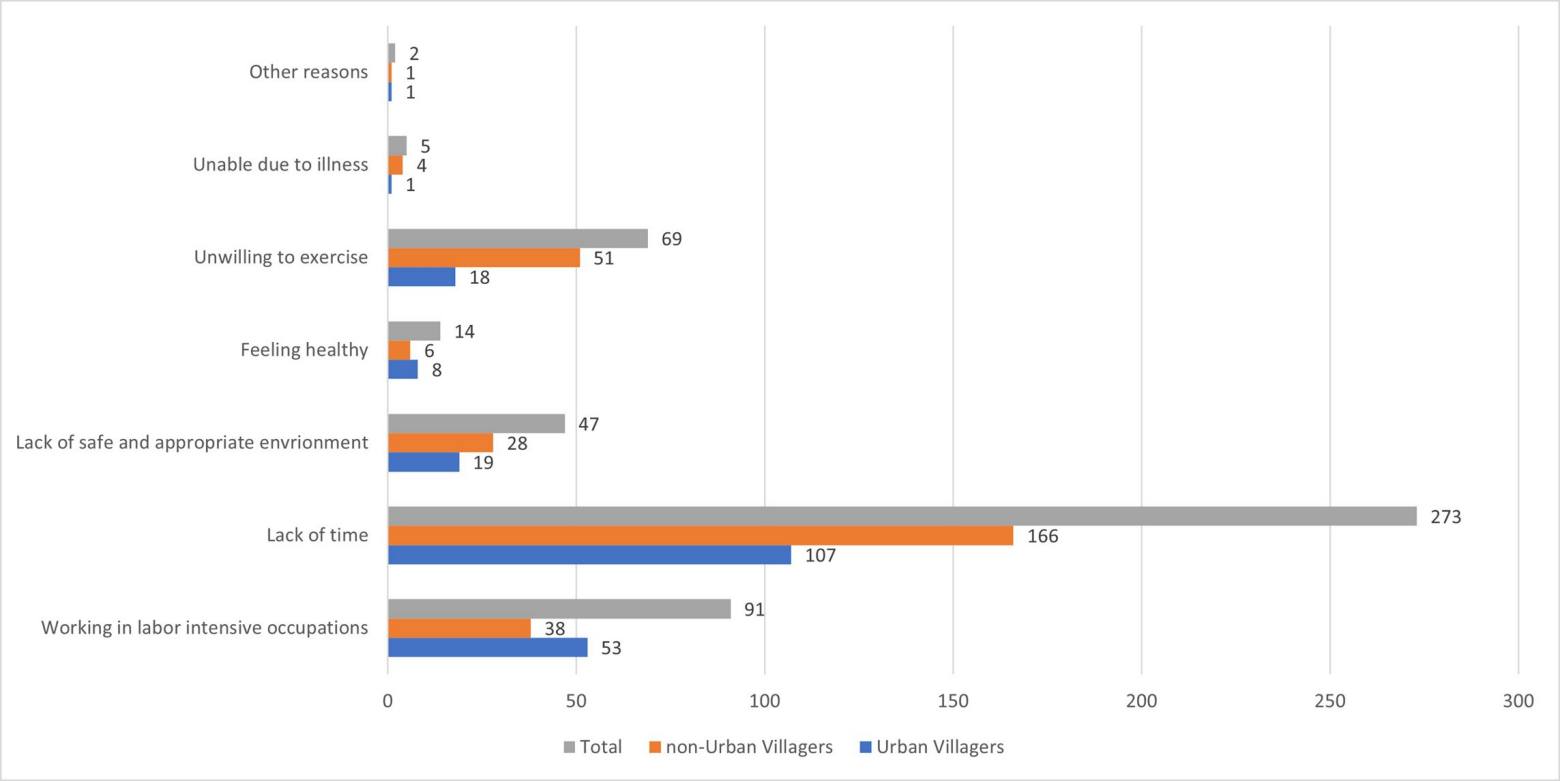
Reasons for not engaging in physical activity among urban villagers and non-urban villagers who engage in physical activity.

Among participants who engage in recreational physical activity, many indicated that no time to exercise as the main reason why they did not engage in physical activity (n = 273) as shown in Fig 2. The second top reasons participants selected as the reasons for not engaging in recreation physical activity was no need to exercise due to labor intensive occupation (n = 91), follow by unwilling to exercise and no place to exercise (n = 69). Some participants also respond that they did not engage in recreation physical activity due to feeling healthy (n = 14) and no need to exercise and unable to engage in recreational physical activity due to illness (n = 5).

When stratified by urban village status, lack of time is the most cited reason for not engaging in physical activity for both urban villagers (n = 107) and non-urban villagers (n = 166). There were more urban villagers (n = 58) compared to non-urban villagers expressed that they do not need to engage in physical activity due to occupations being labor intensive. There were more non-urban villagers (n = 51) expressed that they were unwilling to engage in physical activity than urban villagers (n = 18). Also, higher number of non-urban villagers (n = 28) reported not having appropriate places and/or environments for physical activity compared to urban villagers (n = 19).

## Discussion

The purpose of this secondary data analysis is to determine and compare the prevalence of physical activity engagement among the special population of Chinese urban villagers and non-urban villagers. Both the unadjusted and adjusted logistic regression identified that urban villagers are more likely to engage in recreational physical activity than their counterpart of non-urban villagers. No significant relationship was found between the frequency of engagement in recreational physical activity and urban village status. The multinomial logistic regression also found no significant relationship between hours spend in sedentary behaviors and urban village status. Descriptive analysis shown that both urban villagers and non-urban villagers shared reasons for not engaging in recreational physical activity, such as lack of time to exercise. However, more urban villagers indicated that their labor-intensive occupations are sufficient enough for physical activity. While more non- urban villagers indicated that they are more unwilling to exercise and there are no appropriate places and/or environments for recreational physical activity.

While both urban villagers and non-urban villagers live in urban and well-developed area, the levels of engagement in recreational physical activity were different between the two groups. The results demonstrated that even within the same city, engagement in recreational physical activity could be different by social characteristics. Urban villagers, like non-urban villagers, have access to different public physical activity facilities within the urban area. Physical activity facilities such as parks, sidewalks, and outside of the urban villages are facilities that urban villagers have access to. This is supported by the results that less urban villagers indicated that there are a lack of appropriate places and/or environments for recreational physical activity in compared to non-urban villagers. The ability of utilizing free public physical activity facilities increase the opportunities for urban villagers to engage in recreational physical activity. Having these opportunities allow for urban villagers to obtain a healthier lifestyle of regularly engagement in recreational of physical activity. While it has been found that lower-income neighborhoods, such as urban villages, have less commercial physical activity-related facilities [27]. The results of this study was different from the study conducted by Ortiz-Hernández and Ramos-Ibáñez [28], where they found that Mexican adults living in urban localities and cities with low socio-economic status had a lower probability of engaging in physical activity. However, it is difficult to compare results across different countries as culture and environments are widely different between the countries. Therefore, it is not appropriate to compare the results between the studies. Studies conducted in the US [29] and in the Europe [30] found similar results of adults living in rural areas less likely to engage in physical activity and other psychosocial factors could influence physical activity behaviors. These highlight that there is a need of global effort to promote physical activity in various countries. Further, due to the unique situation of urban village in China, where the housing is surrounded by well-developed buildings and infrastructures, urban villagers have easy access to these different infrastructures.

Income status could potentially be one of the factors explaining the different proportion of urban villagers and non-urban villagers in engagement of recreational physical activity. Individuals living in urban village are more likely to be individuals with lower economic status. Many of these individuals chose to reside in urban village due to the cheap accommodation [31, 32]. Further, many of these individuals might held lower wages and labor-intensive occupations. As evidence by the results of reasons for not engaging recreational physical activity, more urban villagers reported that their occupations are labor intensive enough that either they are too tired to engage in additional physical activity or they felt that they do not need to engage in additional physical activity. This aligned with previous study finding that more rural adults in China engage in work-related physical activity than urban adults [9]. In comparison to urban villagers, fewer participants in the non-urban village group reporting their occupations are too physically demanding that they felt that engagement in recreational physical activity is not necessary. Non-urban villagers are more likely to held office-related occupations, therefore, it limits their ability to engage in physical activity. Past studies had demonstrated that officer workers are more likely to engage in less physical activity and more sedentary behaviors [33]. Further, non-urban villagers might be more likely to have better technology access than urban villagers. Technology such as television and media are found to be associated with lower physical activity levels and high sedentary behavior [34, 35]. This might relate to the higher number of non-Urban Villagers reporting unwilling to engage in recreational physical activity. It is also surprising to find that there are higher numbers of non-urban villagers indicating that the reason for not engaging in recreational physical activity was lack of appropriate places and/or environments. Being consistent with previous research by Munter et al. [9] where Chinese urban adults are less likely to engage in physical activity than Chinese adults with lower economic status living in rural area.

Based on the results of this study, more tailed intervention is needed for Chinese adults not living in urban villages. Even though urban villagers are more likely to be in poor health due to poor housing situation [36, 37], they are more likely to engage in recreational physical activity than non-urban villagers. While the two groups have large number of participants reporting lack of time to engage in recreational physical activity, different interventions should be developed for the two groups. Due to differences in living situations, economic status, and occupations, different reactions and responses to interventions might be different between urban villagers and non-urban villagers. When designing physical activity interventions, there is a need to consider demographic characteristics and socioeconomic factors. For urban villagers, tailed interventions are needed to target group of individuals that believe that physical activity performed during their job are sufficient enough for health. Multiple studies had demonstrated that leisure time physical activity and recreational physical activity are associated with better health quality of life [38–40]. Occupational-related physical activity is not considered to be recreation or leisure physical activity. Therefore, specific interventions are needed targeting urban villagers. Developing interventions in targeting these reasons and solving these barriers for non-urban villagers will be important step for increase the proportion of non-urban villagers in engaging in recreational physical activity. For example, Gu et al. [41] found change in physical activity among office workers after the implementation of a worksite intervention programs at 17 worksites in the urban city of Shanghai with pedometers for 100 days. The goal of using and developing physical activity interventions are to promote recreational physical activity levels among both urban villagers and non-urban villagers.

Further research and studies are warrants in determine the physical activity levels among urban villagers and non-urban villagers. Study had done in the past to examine the physical activity levels of Chines adults [9, 13, 42], but there is a lack of empirical evidence on the physical activity levels of urban villagers. Using additional techniques, such as accelerometers, to collected more detailed data could increase our understanding of physical activity levels of urban villagers. More detailed data such as minutes spend in each intensity of physical activity or number of steps taken each day can better represent the physical activity levels of urban villagers. It has been proposed that an intersectionality approach should be taken when measuring and discussing physical activity levels [43–45]. The interacting factors could provide more detail information on the physical activity of special population such as urban villagers. Often, urban villagers might be considered as individuals living in urban area. However, due to the unique situation of urban village, they are considered a special population living in the urban area. This study demonstrated that there is a need to examine the physical activity levels of special populations living in China. As shown in this study, the proportion of urban villagers and non-urban villagers engaging in recreational physical activity is different, so more research is needed. This data could further facilitate the development of physical activity intervention targeting urban villagers and non-urban villagers. Future researches should also focus on urban villagers and non-urban villagers in meeting physical activity guidelines by the World Health Organization [21]. The current physical activity guidelines for adults over the ages of 18 years old is at least 150 minutes of moderate-to-vigorous physical activity or 75 minutes of vigorous physical activity per week. Examining the prevalence of urban villagers and non-urban villagers in meeting these physical activity guidelines could increase our understanding of the physical activity behaviors and dose-response relationship between physical activity and health among these populations. It is important to note that there is a lack of national and regional physical activity guidelines in China [46]. Developing these physical activity guidelines could be beneficial for Chinese citizen as there is a guideline for them to follow.

One interesting find of the analysis was that education might have an influence on physical activity-related outcomes among urban villagers and non-urban villagers. Based on the adjusted logistic regression model, in compare to no formal education and only completing primary education, other education levels (i.e., middle school, high school, professional college and university) are less likely to engage in physical activity. The analysis also found that higher education is associated with longer time spent in sedentary behaviors. The results align with previous study examining the decline of physical activity levels among Chinese adults [14]. The study found that the greater availability of higher educational institutions is strongly associated with the declines of physical activities based on data from the 1991–2006 China Health and Nutrition Surveys [47]. Individuals with higher education are more likely to have office-related positions. Officer workers are more likely to spend more time in sedentary behaviors [48]. In addition, it was found that Chinese adults who completed high school education are less likely to engage in occupational-related physical activity [9]. These results suggested that physical activity interventions are needed for individuals with higher education. To ensure that physical activity become a lifelong habit among Chinese adults, there is need to develop physical activity intervention targeting adults at various educational levels. For example, requiring physical education or physical activity classes for students in middle schools, high schools, and colleges and universities. Requirement of physical education in early childhood is positively associated with physical activity levels in adulthood [49]. Individuals who had taken a physical activity course while in colleges and universities report higher physical activity levels in adulthood compared to those that did not take a physical activity course [50]. Continuation promotion of physical activity through various different educational institutions could potentially increase physical activity levels of adults.

### Limitation

To the authors’ knowledge, this is the first of the few studies that examined the physical activity levels of urban villagers in China. The strength of this study is including the special population of urban villagers. However, this study is not without its limitation. The data used in the analysis are based on self-reported data. There could be potential recall and social bias. These biases could lead to misclassification of data and results [51]. In addition to biases, there could be low generalizability of the results. Due to the data only included participants living in the Luohu, Shenzhen, China, the results might be only generalized to this particular populations living in Shenzhen. However, it is assumed that urban villagers across China shared the similar characteristics of lower economic status, migrant workers, labor intensive worker, poor living situation, and lack of infrastructures. It is important to note that the survey did not utilized the International Physical Activity Questionary (IPAQ) in the surveillance system. This could lead to misunderstanding of questions by the participants. To limit misunderstanding, all data collected were in Chinese via face-to-face interview by trained personals.

## Conclusion

Overall, the proportion of urban villagers and non-urban villagers in engaging in recreational physical activity are different with urban villagers more likely to engage in recreational physical activity. While participants from both groups expressed that lack of time as a barrier in engaging in recreational physical activity, non-urban villagers are more likely to reported that they are unwilling to participate in recreational physical activity and lack appropriate place and/environment for recreational physical activity. Urban villagers are more likely to reported that they do not engage in recreational physical activity due to work-related physical activity. Physical activity interventions are needed to target these various barriers in preventing urban villagers and non-urban villagers in participating from recreational physical activity. Further research is warranted in order to better understanding the physical activity levels of the special population of urban villagers living in China.

## Supporting information

S1 Table Odd ratios of urban villagers and non-urban villagers in engaging in recreational physical activity: Adjusted model outcomes. (DOCX)

S2 Table Odd ratios of frequency of engaging in recreational physical activity per week between urban villagers and non-urban villagers: Adjusted model outcomes. (DOCX)

S3 Table Odd ratios of average hours spend in sedentary behaviors per day between urban villagers and non-urban villagers. (DOCX)

S1 File Questionnaire Chinese. (DOCX)

S2 File Questionnaire English. (DOCX)

## References

[1] Haskell WL, Blair SN, Hill JO. Physical activity: Health outcomes and importance for public health policy. Preventive Medicine. 2009 Oct 1;49(4):280–2. doi: 10.1016/j.ypmed.2009.05.002 19463850

[2] Steinbeck KS. The importance of physical activity in the prevention of overweight and obesity in childhood: a review and an opinion. Obesity Reviews. 2001;2(2):117–30. doi: 10.1046/j.1467-789x.2001.00033.x 12119663

[3] Warburton DER, Nicol CW, Bredin SSD. Health benefits of physical activity: the evidence. Canadian Medical Association Journal. 2006 Mar 14;174(6):801–9. doi: 10.1503/cmaj.051351 16534088 PMC1402378

[4] Eime RM, Young JA, Harvey JT, Charity MJ, Payne WR. A systematic review of the psychological and social benefits of participation in sport for adults: informing development of a conceptual model of health through sport. Int J Behav Nutr Phys Act. 2013 Dec 7;10:135. doi: 10.1186/1479-5868-10-135 24313992 PMC4028858

[5] Singh MAF. Exercise to prevent and treat functional disability. Clin Geriatr Med. 2002 Aug;18(3):431–62, vi–vii. doi: 10.1016/s0749-0690(02)00016-2 12424867

[6] Tak E, Kuiper R, Chorus A, Hopman-Rock M. Prevention of onset and progression of basic ADL disability by physical activity in community dwelling older adults: A meta-analysis. Ageing Research Reviews. 2013 Jan 1;12(1):329–38. doi: 10.1016/j.arr.2012.10.001 23063488

[7] World Health Organization. WHO | Physical Inactivity: A Global Public Health Problem [Internet]. WHO. World Health Organization; 2020 [cited 2020 Sep 22]. Available from: https://www.who.int/dietphysicalactivity/factsheet_inactivity/en/.

[8] Li F. Physical activity and health in the presence of China’s economic growth: Meeting the public health challenges of the aging population. Journal of Sport and Health Science. 2016 Sep 1;5(3):258–69. doi: 10.1016/j.jshs.2016.06.004 30356539 PMC6188738

[9] Muntner P, Gu D, Wildman RP, Chen J, Qan W, Whelton PK, et al. Prevalence of Physical Activity Among Chinese Adults: Results From the International Collaborative Study of Cardiovascular Disease in Asia. Am J Public Health. 2005 Sep 1;95(9):1631–6. doi: 10.2105/AJPH.2004.044743 16051938 PMC1449408

[10] Zhang L, Wang Z, Wang X, Chen Z, Shao L, Tian Y, et al. Prevalence of overweight and obesity in China: Results from a cross-sectional study of 441 thousand adults, 2012–2015. Obesity Research & Clinical Practice. 2020 Mar 1;14(2):119–26. 32139330 10.1016/j.orcp.2020.02.005

[11] Natinal Center for Cardiovascular Disease. China. Report on Cardiovascular Diseases in China 2017. Beijing: Encyclopedia of China Publishing House; 2018.

[12] Liu L. Rural-Urban Disparities in Cardiovascular Disease Mortality Among Middle-Age Men in China. Asia Pac J Public Health. 2020 Sep 11;1010539520956446. doi: 10.1177/1010539520956446 32917101

[13] Peters TM, Moore SC, Xiang YB, Yang G, Shu XO, Ekelund U, et al. Accelerometer-Measured Physical Activity in Chinese Adults. American Journal of Preventive Medicine. 2010 Jun 1;38(6):583–91. doi: 10.1016/j.amepre.2010.02.012 20494234 PMC2897243

[14] Chen M, Wu Y, Narimatsu H, Li X, Wang C, Luo J, et al. Socioeconomic Status and Physical Activity in Chinese Adults: A Report from a Community-Based Survey in Jiaxing, China. PLOS ONE. 2015 Jul 15;10(7):e0132918. doi: 10.1371/journal.pone.0132918 26177205 PMC4503452

[15] Shi Z, Lien N, Kumar BN, Holmboe-Ottesen G. Physical activity and associated socio-demographic factors among school adolescents in Jiangsu Province, China. Preventive Medicine. 2006 Sep 1;43(3):218–21. doi: 10.1016/j.ypmed.2006.04.017 16762405

[16] Liu Y, He S. Chinese Urban Villages as Marginalized Neighbourhoods under Rapid Urbanization. In: Wu F, Webster C, editors. Marginalization in Urban China: Comparative Perspectives [Internet]. London: Palgrave Macmillan UK; 2010 [cited 2020 Oct 2]. p. 177–200. (International Political Economy Series). Available from: 10.1057/9780230299122_10

[17] Liu Y, He S, Wu F, Webster C. Urban villages under China’s rapid urbanization: Unregulated assets and transitional neighbourhoods. Habitat International. 2010 Apr 1;34(2):135–44.

[18] Zhang L, Zhao SXB, Tian JP. Self-help in housing and chengzhongcun in China’s urbanization. International Journal of Urban and Regional Research. 2003;27(4):912–37.

[19] Shi L, Patil VP, Leung W, Zheng Q. Willingness to use and satisfaction of primary care services among locals and migrants in Shenzhen, China. Health & Social Care in the Community [Internet]. [cited 2021 Jul 7];n/a(n/a). Available from: https://onlinelibrary.wiley.com/doi/abs/10.1111/hsc.13418 33978287 10.1111/hsc.13418

[20] Yang X. Research on the Optimization of Social Sports Participation Path in “Urban Villages.” In 2018 [cited 2020 Oct 23]. Available from: https://kns.cnki.net/KCMS/detail/detail.aspx?dbcode=IPFD&dbname=IPFDLAST2018&filename=LRCM201802002061&v=MTc1OTRiS0lGc1hLVC9JWTdHNEg5bk1yWTlGWnVzSkRSTkt1aGRobmo5OFRuanFxeGRFZU1PVUtyaWZaZVp1RmluZ1Vy.

[21] World Health Organization. Global recommendations on physical activity for health. Geneva: World Health Organization; 2010. 26180873

[22] Bork T, Kraas F, Xue D, Li Z. Urban environmental health challenges in China’s villages-in-the-city. Geographische Zeitschrift. 2011;99(1):16–35.

[23] Jiang J, Wang P. Health status in a transitional society: urban-rural disparities from a dynamic perspective in China. Population Health Metrics. 2018 Dec 27;16(1):22. doi: 10.1186/s12963-018-0179-z 30591053 PMC6307183

[24] Duan J, Cao D. Investigation on the Status Quo of Mass Sports Participation in Urban Villages——Taking Wanbolin District of Taiyuan City as an Example [Internet]. North University of China; 2017 [cited 2020 Oct 23]. Available from: https://kns.cnki.net/KCMS/detail/detail.aspx?dbcode=CJFQ&dbname=CJFDLAST2017&filename=TYGJ201703008&v=MDMxOTk4ZVgxTHV4WVM3RGgxVDNxVHJXTTFGckNVUjdxZVorWnJGaUhuVnIvTE1UVE1aTEc0SDliTXJJOUZiSVI=.

[25] Long H, Li Y, Yu H, Yang W, Wu M. Research on Youth Sports Participation in Urban Villages of Kunming City in the Process of Urbanization. Contemporary Sports Technology. 2019;9(06):167–8.

[26] Chen X. The Problems Research of Griddization Management in Longgang District of Shenzhen City [Internet] [Master]. Central China Normal University; 2015 [cited 2020 Oct 24]. Available from: https://gb.oversea.cnki.net/kcms/detail/detail.aspx?recid=&FileName=1016038151.nh&DbName=CMFD201602&DbCode=CMFD.

[27] Powell LM, Slater S, Chaloupka FJ, Harper D. Availability of Physical Activity–Related Facilities and Neighborhood Demographic and Socioeconomic Characteristics: A National Study. Am J Public Health. 2006 Sep;96(9):1676–80. doi: 10.2105/AJPH.2005.065573 16873753 PMC1551946

[28] Ortiz-Hernández L, Ramos-Ibáñez N. Sociodemographic factors associated with physical activity in Mexican adults. Public Health Nutrition. 2010 Jul;13(7):1131–8. doi: 10.1017/S1368980010000261 20196912

[29] Wilcox S, Castro C, King AC, Housemann R, Brownson RC. Determinants of leisure time physical activity in rural compared with urban older and ethnically diverse women in the United States. Journal of Epidemiology & Community Health. 2000 Sep 1;54(9):667–72. doi: 10.1136/jech.54.9.667 10942445 PMC1731735

[30] Moreno-Llamas A, García-Mayor J, De la Cruz-Sánchez E. Urban-rural differences in trajectories of physical activity in Europe from 2002 to 2017. Health & Place. 2021 May 1;69:102570. doi: 10.1016/j.healthplace.2021.102570 33873131

[31] Keung Wong DF, Li CY, Song HX. Rural migrant workers in urban China: living a marginalised life: Rural migrant workers in urban China. International Journal of Social Welfare. 2007 Jan;16(1):32–40.

[32] Lu Z, Song S. Rural–urban migration and wage determination: The case of Tianjin, China. China Economic Review. 2006 Jan;17(3):337–45.

[33] Parry S, Straker L. The contribution of office work to sedentary behaviour associated risk. BMC Public Health. 2013 Apr 4;13:296. doi: 10.1186/1471-2458-13-296 23557495 PMC3651291

[34] Harris JL, Bargh JA. The Relationship between Television Viewing and Unhealthy Eating: Implications for Children and Media Interventions. Health Commun. 2009 Oct;24(7):660–73. doi: 10.1080/10410230903242267 20183373 PMC2829711

[35] Keadle SK, Arem H, Moore SC, Sampson JN, Matthews CE. Impact of changes in television viewing time and physical activity on longevity: a prospective cohort study. International Journal of Behavioral Nutrition and Physical Activity. 2015 Dec 18;12(1):156. doi: 10.1186/s12966-015-0315-0 26678502 PMC4683741

[36] Gao Y, Shahab S, Ahmadpoor N. Morphology of Urban Villages in China: A Case Study of Dayuan Village in Guangzhou. Urban Science. 2020 May 7;4(2):23.

[37] Wu F. Housing in Chinese Urban Villages: The Dwellers, Conditions and Tenancy Informality. Housing Studies. 2016 Oct 2;31(7):852–70.

[38] Tessier S, Vuillemin A, Bertrais S, Boini S, Le Bihan E, Oppert J-M, et al. Association between leisure-time physical activity and health-related quality of life changes over time. Preventive Medicine. 2007 Mar 1;44(3):202–8. doi: 10.1016/j.ypmed.2006.11.012 17208289

[39] Vuillemin A, Boini S, Bertrais S, Tessier S, Oppert J-M, Hercberg S, et al. Leisure time physical activity and health-related quality of life. Preventive Medicine. 2005 Aug 1;41(2):562–9. doi: 10.1016/j.ypmed.2005.01.006 15917053

[40] Wendel-Vos GCW, Schuit AJ, Tijhuis MAR, Kromhout D. Leisure time physical activity and health-related quality of life: Cross-sectional and longitudinal associations. Qual Life Res. 2004 Apr 1;13(3):667–77. doi: 10.1023/B:QURE.0000021313.51397.33 15130029

[41] Gu M, Wang Y, Shi Y, Yu J, Xu J, Jia Y, et al. Impact of a group-based intervention program on physical activity and health-related outcomes in worksite settings. BMC Public Health. 2020 Jun 15;20(1):935. doi: 10.1186/s12889-020-09036-2 32539787 PMC7294670

[42] Zhu W, Chi A, Sun Y. Physical activity among older Chinese adults living in urban and rural areas: A review. Journal of Sport and Health Science. 2016 Sep 1;5(3):281–6. doi: 10.1016/j.jshs.2016.07.004 30356525 PMC6188614

[43] Abichahine H, Veenstra G. Inter-categorical intersectionality and leisure-based physical activity in Canada. Health Promot Int. 2017 Aug 1;32(4):691–701. doi: 10.1093/heapro/daw009 26976822

[44] Herrick SSC, Duncan LR. A Qualitative Exploration of LGBTQ+ and Intersecting Identities Within Physical Activity Contexts. Journal of Sport and Exercise Psychology. 2018 Dec 1;40(6):325–35. doi: 10.1123/jsep.2018-0090 30537884

[45] Ray R. An Intersectional Analysis to Explaining a Lack of Physical Activity Among Middle Class Black Women. Sociology Compass. 2014;8(6):780–91.

[46] Xu J, Gao C. Physical activity guidelines for Chinese children and adolescents: The next essential step. J Sport Health Sci. 2018 Jan;7(1):120–2. doi: 10.1016/j.jshs.2017.07.001 30356441 PMC6180532

[47] Ng SW, Norton EC, Popkin BM. Why have physical activity levels declined among Chinese adults? Findings from the 1991–2006 China health and nutrition surveys. Social Science & Medicine. 2009 Apr 1;68(7):1305–14. doi: 10.1016/j.socscimed.2009.01.035 19232811 PMC2731106

[48] Clemes SA, O’Connell SE, Edwardson CL. Office Workers’ Objectively Measured Sedentary Behavior and Physical Activity During and Outside Working Hours. Journal of Occupational and Environmental Medicine. 2014 Mar;56(3):298–303. doi: 10.1097/JOM.0000000000000101 24603203

[49] Trudeau F, Laurencelle L, Shephard RJ. Tracking of physical activity from childhood to adulthood. Med Sci Sports Exerc. 2004 Nov;36(11):1937–43. doi: 10.1249/01.mss.0000145525.29140.3b 15514510

[50] Sparling PB, Snow TK. Physical activity patterns in recent college alumni. Res Q Exerc Sport. 2002 Jun;73(2):200–5. doi: 10.1080/02701367.2002.10609009 12092895

[51] Althubaiti A. Information bias in health research: definition, pitfalls, and adjustment methods. J Multidiscip Healthc. 2016 May 4;9:211–7. doi: 10.2147/JMDH.S104807 27217764 PMC4862344

