# Supplementary material for: Level of engagement of recreational physical activity of urban villagers in Luohu, Shenzhen, China
Source: PLoS One. 2021 Oct 28;16(10):e0258085. doi: 10.1371/journal.pone.0258085 (PMC8553036; doi:10.1371/journal.pone.0258085)
Supplement: S1 Table — (DOCX) [file pone.0258085.s001.docx]

| S1 Table. Odd Ratios of urban villagers and non-urban villagers in engaging in recreational physical activity: adjusted model outcomes | | |
| --- | --- | --- |
| Variables | Engagement in recreational physical activity | |
|  | OR | 95%CI |
| Constant | 2.26 | 0.56, 9.10 |
| Urban village | 1.90* | 1.40, 2.57 |
| Gender | 1.15 | 0.81, 1.62 |
| Age | 0.99 | 0.98, 1.01 |
| Employment Status | 1.04 | 0.71, 1.53 |
| Education |  |  |
| Professional college, and university | 0.26* | 0.15, 0.45 |
| High school | 0.29* | 0.18, 0.48 |
| Middle school | 0.53* | 0.34, 0.83 |
| No education & primary | ref. |  |
| Marriage | 1.26 | 0.87, 1.82 |
| Household registration (Hukou) | 0.67* | 0.46, 0.96 |
| BMI | 0.95* | 0.91, 0.99 |
| Central obesity | 1.87 | 0.89, 3.94 |
| Hypertension | 1.43 | 0.81, 2.55 |
| Diabetes | 0.41 | 0.12, 1.37 |
| Smoke | 1.35 | 0.93, 1.98 |

Abbreviations: OR, odds ratio; CI, confidence interval.

^a^Boldfaced numerals indicate p-value <0.05.
