# Supplementary material for: Level of engagement of recreational physical activity of urban villagers in Luohu, Shenzhen, China
Source: PLoS One. 2021 Oct 28;16(10):e0258085. doi: 10.1371/journal.pone.0258085 (PMC8553036; doi:10.1371/journal.pone.0258085)
Supplement: S2 Table — (DOCX) [file pone.0258085.s002.docx]

| S2 Table. Odd ratios of frequency of engaging in recreational physical activity per week between urban villagers and non-urban villagers: adjusted model outcomes | | | | | | | | |
| --- | --- | --- | --- | --- | --- | --- | --- | --- |
| Variables | 1 – 2 times vs. < 1 time (ref.) | |  | 3 – 5 times vs. < 1 time (ref.) | |  | > 6 times vs. < 1 time (ref.) | |
|  | AOR | 95%CI |  | AOR | 95%CI |  | AOR | 95%CI |
| Constant | 6.66 | 0.14, 328.10 |  | 1.34 | 0.03, 68.84 |  | 0.33 | 0.00, 23.98 |
| Urban village | 1.07 | 0.44, 2.64 |  | 0.98 | 0.39, 2.43 |  | 0.83 | 0.32, 2.15 |
| Gender | 1.21 | 0.53, 2.76 |  | 0.95 | 0.41, 2.20 |  | 1.38 | 0.57, 3.37 |
| Age | 1.03 | 0.98, 1.08 |  | **1.06*** | **1.01, 1.12** |  | **1.10*** | **1.05, 1.16** |
| Employment Status | 0.37 | 0.08, 1.66 |  | **0.23*** | **0.05, 1.05** |  | **0.18*** | **0.04, 0.84** |
| Education |  |  |  |  |  |  |  |  |
| Professional college, and university | 2.05 | 0.35, 12.14 |  | 1.64 | 0.28, 9.56 |  | 1.60 | 0.26, 9.98 |
| High school | 1.36 | 0.25, 7.42 |  | 1.01 | 0.19, 5.39 |  | 1.58 | 0.28, 8.83 |
| Middle school | 1.84 | 0.30, 11.12 |  | 1.91 | 0.32, 11.29 |  | 3.13 | 0.51, 19.23 |
| No education & primary | ref. |  |  |  |  |  |  |  |
| Marriage | 1.01 | 0.41, 2.47 |  | 0.77 | 0.31, 1.91 |  | 0.72 | 0.28, 1.90 |
| Household registration (Hukou) | 0.55 | 0.22, 1.37 |  | **0.36*** | **0.14, 0.91** |  | **0.28*** | **0.11, 0.74** |
| BMI | 0.97 | 0.87, 1.09 |  | 1.03 | 0.93, 1.15 |  | 1.03 | 0.92, 1.16 |
| Central obesity | 0.99 | 0.11, 9.07 |  | 1.25 | 0.14, 11.45 |  | 0.67 | 0.04, 11.22 |
| Hypertension | 1.51 | 0.17, 13.28 |  | 2.15 | 0.25, 18.25 |  | 2.28 | 0.26, 19.82 |
| Diabetes | 0.64 | 0.07, 6.15 |  | 0.99 | 0.11, 8.93 |  | 0.24 | 0.02, 3.07 |
| Smoke | 0.80 | 0.34, 1.92 |  | 0.69 | 0.28, 1.67 |  | 0.77 | 0.30, 2.00 |

Abbreviations: OR, odds ratio; CI, confidence interval.

^a^Boldfaced numerals indicate p-value <0.05.
