# Supplementary material for: Level of engagement of recreational physical activity of urban villagers in Luohu, Shenzhen, China
Source: PLoS One. 2021 Oct 28;16(10):e0258085. doi: 10.1371/journal.pone.0258085 (PMC8553036; doi:10.1371/journal.pone.0258085)
Supplement: S3 Table — (DOCX) [file pone.0258085.s003.docx]

| S3 Table. Odd ratios of average hours spend in sedentary behaviors per day between urban villagers and non-urban villagers | | | | | | | | | | | |
| --- | --- | --- | --- | --- | --- | --- | --- | --- | --- | --- | --- |
|  | 2 – 4 hours vs. < 2 hours | |  | 5 – 8 hours vs. < 2 hours | |  | 9 – 12 hours vs. < 2 hours | |  | >12 hours vs. < 2 hours | |
| Variables | AOR | 95%CI |  | AOR | 95%CI |  | AOR | 95%CI |  | AOR | 95%CI |
| Constant | 0.58 | 0.09, 3.87 |  | 0.40 | 0.07, 2.36 |  | 0.60 | 0.07, 5.41 |  | 0.06 | 0.00, 2.07 |
| Urban village | 0.85 | 0.58, 1.25 |  | 1.18 | 0.79, 1.75 |  | 1.43 | 0.86, 2.38 |  | 0.77 | 0.35, 1.71 |
| Gender | 1.02 | 0.66, 1.56 |  | 0.99 | 0.64, 1.54 |  | 1.34 | 0.78, 2.32 |  | 1.50 | 0.63, 3.55 |
| Age | 1.00 | 0.98, 1.02 |  | 1.01 | 0.99, 1.03 |  | 0.97* | 0.95, 1.00 |  | 0.98 | 0.95, 1.03 |
| Employment Status | 1.20 | 0.75, 1.92 |  | 1.15 | 0.70, 1.86 |  | 1.14 | 0.62, 2.10 |  | 1.14 | 0.43, 3.05 |
| Education |  |  |  |  |  |  |  |  |  |  |  |
| Professional college, and university | 2.97* | 1.49, 5.93 |  | 3.01* | 1.51, 6.00 |  | 5.40* | 2.12, 13.77 |  | 6.52* | 1.54, 27.69 |
| High school | 2.80* | 1.55, 5.08 |  | 1.97* | 1.09, 3.58 |  | 1.86 | 0.77, 4.50 |  | 2.37 | 0.59, 9.48 |
| Middle school | 1.58 | 0.90, 2.75 |  | 1.05 | 0.59, 1.84 |  | 1.44 | 0.62, 3.36 |  | 1.34 | 0.34, 5.35 |
| No education & primary | ref. |  |  | ref. |  |  | ref. |  |  | ref. |  |
| Marriage | 0.81 | 0.50, 1.32 |  | 0.47* | 0.29, 0.76 |  | 0.69 | 0.39, 1.23 |  | 0.54 | 0.24, 1.21 |
| Household registration (Hukou) | 1.27 | 0.80, 2.02 |  | 1.48 | 0.91, 2.39 |  | 2.08* | 1.16, 3.71 |  | 1.24 | 0.54, 2.85 |
| BMI | 1.04 | 0.99, 1.10 |  | 1.03 | 0.97, 1.09 |  | 1.00 | 0.93, 1.23 |  | 1.06 | 0.96, 1.16 |
| Central obesity | 0.31 | 0.07, 1.34 |  | 0.65 | 0.27, 1.57 |  | 0.63 | 0.22, 1.74 |  | 0.43 | 0.04, 4.35 |
| Hypertension | 0.85 | 0.40, 1.77 |  | 1.26 | 0.62, 2.55 |  | 1.77 | 0.71, 4.42 |  | 0.95 | 0.20, 4.61 |
| Diabetes | 0.35 | 0.10, 1.23 |  | 0.40 | 0.12, 1.33 |  | 0.62 | 0.15, 2.56 |  | 0.69 | 0.08, 6.30 |
| Smoke | 0.80 | 0.49, 1.28 |  | 0.96 | 0.59, 2.36 |  | 1.36 | 0.75, 2.49 |  | 2.70* | 1.13, 6.45 |

Abbreviations: OR, odds ratio; CI, confidence interval.

^a^Boldfaced numerals indicate p-value <0.05.
