# Supplementary material for: Level of engagement of recreational physical activity of urban villagers in Luohu, Shenzhen, China
Source: PLoS One. 2021 Oct 28;16(10):e0258085. doi: 10.1371/journal.pone.0258085 (PMC8553036; doi:10.1371/journal.pone.0258085)
Supplement: S1 File — (DOCX) [file pone.0258085.s004.docx]

罗湖区居民社区卫生诊断调查表

| 表1. 家庭一般情况调查表 | | |
| --- | --- | --- |
| 表由最熟悉家庭情况的人回答 | | code |
| 1 | 近半年内，您家常住人口数是多少？（包括亲戚、保姆等，但不包括外出半年以上的家庭成员） | f1 |
| 2 | 其中有几人户口是深圳户籍？ | f2 |
| 3 | 您家住房类型是下列的哪种类型？①自建楼房 ②普通商品房 ③与他人合租或集体宿舍 | f3 |
| 4 | 您居住小区环境卫生 ①好 ②中 ③差 | f4 |
| 5 | 您家是否经常开窗通风？ ①每周通风0-2次 （差） ②一周通风3-5次（中） ③每周5次以上（好） | f5 |
| 6 | 一天中，您家中卧室是否有太阳光照？ ①是 ②否 | f6 |
| 7 | 您家4-10月是否有蚊子、苍蝇？ ①没有（好） ②每日蚊子或苍蝇1-2只（中） ③每日蚊子或苍蝇2只以上（差） | f7 |
| 8 | 您家是否安装纱窗？ ①是 ②否 | f8 |
| 9 | 您家是否经常对碗筷进行消毒？ ①是（每周1次以上） ②否 | f9 |
| 10 | 您家是否备有以下常用药品？（可多选） ①感冒药 ②外伤处理用药（双氧水、碘酊，红药水等）③常用医疗器械（镊子，棉签，纱布，绷带，创可贴） ④胃药、肠道用药 ⑤皮肤用药（地塞米松乳膏，莫匹罗星软膏等） ⑥测量工具（体温计 血压仪） ⑦其他 | f10 |
|  |  |  |
|  |  |  |
|  |  |  |
|  |  |  |
|  |  |  |
|  |  |  |
|  |  |  |
|  |  |  |
| 11 | 过去一年内，您的家庭用于医疗服务及医疗用品的全部支出为多少元?(包括自费和可报销医疗费用，但不包括保健食品、保健用品费用) ＿＿＿元 | f11 |
| 12 | 其中，自费医疗费用支出为多少元?(不包括保健食品、保健用品费用) ＿＿＿＿＿＿元 | f12 |
| 13 | 您家是否采取过限盐措施（主动控制每日食物中的盐量）？①  是 ②否 | f13 |
| 14 | 您家做菜的时候是否采取过控油措施（主动控制每日烹调用的食用油量）吗？①  是 ②否 | f14 |
| 15 | 您家通常在晚上一起就餐的人数？（包括非家庭成员）＿＿＿＿人 | f15 |

| 表2. 家庭成员基本情况调查表 | | |
| --- | --- | --- |
| 表由最熟悉家庭情况的人回答 | | code |
| 1 | 下列调查问题将由谁回答： ①自己回答（请跳至第3题） ②由他人代答 | a1 |
| 2 | 他人代答的原因： ①本人外出 ②年龄太小 ③文化程度低 ④本人头脑不清 ⑤本人不愿意回答 | a2 |
| 3 | 成员年龄：＿＿＿＿＿＿＿＿岁 | a3 |
| 4 | 性别： ① 男 ②女 | a4 |
| 5 | 民族： ① 汉族 ②少数民族 | a5 |
| 6 | 您的户口：①深圳户籍 ②非深户 | a6 |
| 7 | 婚姻状况： ①未婚 ②已婚 ③离婚 ④丧偶 ⑤其它＿＿＿＿ | a7 |
| 8 | 文化程度：①  文盲 ②小学 ③初中 ④高中/中专 ⑤大专 ⑥大学本科 ⑦研究生及以上 | a8 |
| 9 | 职业类型：①行政干部 ②科技人员 ③医务人员 ④工人 ⑤商业/服务业员工 ⑥教师 ⑦金融保险 ⑧学生 ⑨离退休人员 ⑩无业人员 ⑪家庭主妇 ⑫农民 ⑬军人 ⑭个体户 ⑮私营企业主 ⑯自由职业者 ⑰其他＿＿＿＿ | a9 |
| 10 | 您目前参加了哪些医疗保险?（可多选） ①社会基本医疗保险 ②深圳劳务工医疗保险 ③新型农村合作医疗④商业保险 ⑤未参加任何保险 ⑥统筹医疗保险 ⑦少儿医疗保险⑧其他＿＿＿＿＿＿＿＿＿ | a10 |
|  |  |  |
|  |  |  |
|  |  |  |
|  |  |  |
|  |  |  |
|  |  |  |
|  |  |  |
| 11 | 您是否有每年定期体检？ ①是 ②否 | a11 |
| 12 | 您是否患有高血压病？ ①是 ②否（如选“否”请跳至第14题） | a12 |
| 13 | 在哪里诊断的？ ①私人诊所 ②社区卫生服务中心（社康中心） ③区级综合医院 ④市级综合医院 ⑤区级或市级专科医院 ⑥省级以上医院 ⑦其他＿＿＿ | a13 |
| 14 | 您是否患有糖尿病？ ①是 ②否（如选“否”请跳至第16题） | a14 |
| 15 | 在哪里诊断的？ ①私人诊所 ②社区卫生服务中心（社康中心） ③区级综合医院 ④市级综合医院 ⑤区级或市级专科医院 ⑥省级以上医院 ⑦其他＿＿＿ | a15 |
| 16 | 您是否还患有经医生诊断的其他疾病？ （可多选）①  无其他疾病 ②脑卒中 ③慢性支气管炎 ④慢性乙肝 ⑤高血脂症 ⑥白内障 ⑦骨质疏松 ⑧前列腺肥大 ⑨颈椎病 ⑩慢性肠炎 ⑪慢性鼻炎 ⑫肾结石 ⑬精神病 ⑭良性肿瘤 ⑮恶性肿瘤 ⑯冠心病 ⑰哮喘 ⑱其他＿＿ | a16 |
|  |  |  |
|  |  |  |
|  |  |  |
|  |  |  |
|  |  |  |
|  |  |  |
|  |  |  |
|  |  |  |
|  |  |  |
|  |  |  |
|  |  |  |
|  |  |  |
|  |  |  |
|  |  |  |
|  |  |  |
|  |  |  |
|  |  |  |
|  |  |  |
| 17 | 调查前的2周内，您是否觉得有身体不适或患有急、慢性疾病? ①是 ②否（跳至D住院调查） | a17 |
| 18 | 主要有哪些不适？（多选） ①发烧 ②头晕 ③心慌/心悸 ④咳嗽 ⑤腹泻 ⑥腹痛 ⑦皮疹 ⑧外伤 ⑨其他＿＿＿＿＿＿＿＿ | a18 |
|  |  |  |
|  |  |  |
|  |  |  |
|  |  |  |
|  |  |  |
|  |  |  |
|  |  |  |
|  |  |  |
|  |  |  |
| 19 | 您患的是什么病或伤？ ①无医生明确诊断 ②诊断明确(填疾病名称和编码) ＿＿＿＿＿＿＿＿＿＿＿＿＿＿＿＿＿＿＿ | a19 |
| 20 | 您感觉所患病伤的严重程度： ①不严重 ②严重 | a20 |
| 21 | 您这次的病伤是什么时候开始发病的？ ①2周内新发生的 ②急性病2周前发生延续到2周内 ③慢性病持续到2周内或2周内急性发作 | a21 |
| 22 | 本次病伤在调查前2周持续了多少天？ ＿＿＿＿天 | a22 |
| 23 | （如你是学生或已在工作，老年人、没有工作者和没有上学的儿童可不作答），调查前2周内，是否因本次病伤休病假？①否 ②是，休＿＿天 | a23 |
| 24 | 您患病后，是否有采用什么方式进行治疗？①否(跳至28题) ②是，自我医疗否(跳至29题) ③是，到医疗机构看病治疗 ④是，自我医疗和到医疗机构看病治疗 ⑤其他＿＿ | a24 |
| 25 | 两周内，为该病伤第一次就诊是在下列哪类医疗机构？ ①私人诊所 ②社区卫生服务中心（社康中心） ③区级综合医院 ④市级综合医院 ⑤区级或市级专科医院 ⑥省级以上医院 ⑦其他＿＿＿ | a25 |
| 26 | 选择上述单位就诊的主要原因是：（可多选） ①距离近 ②收费合理 ③ 技术水平高 ④设备条件好 ⑤定点单位 ⑥药品丰富 ⑦服务态度好 ⑧有熟人/信赖医生 ⑨其他＿＿＿＿＿ | a26 |
|  |  |  |
|  |  |  |
|  |  |  |
|  |  |  |
|  |  |  |
|  |  |  |
|  |  |  |
|  |  |  |
|  |  |  |
| 27 | 看病后，是否根据医生处方在非就诊医院或药店买药？ ①是 ②否 | a27 |
| 28 | 未治疗的最主要原因？（可多选） ①  自感病轻 ②经济困难 ③无时间 ④交通不便 ⑤医疗服务差 ⑥自觉无有效措施 ⑦其他＿＿ | a28 |
| 29 | 在过去一年内，您是否有因病住院治疗？ ①是 ②否（结束调查） | a29 |
| 30 | 您住院原因：①疾病 ②损伤或中毒 ③康复 ④计划生育 ⑤分娩 ⑥其它＿＿＿＿＿ | a30 |
| 31 | 因疾住院的疾病名称？①(填写疾病名称，请不要填写症状)＿＿＿＿＿＿＿＿＿＿②(填疾病编码) ＿＿＿＿＿＿＿＿＿＿ | a31 |
|  |  |  |
| 32 | 最近一年，您因这种病伤住过几次医院？＿＿＿次 | a32 |
| 33 | 您最近一次住院的医疗机构类型？①区级综合医院 ②市级综合医院③区级或市级专科医院 ④省级以上医院 ⑤其他＿＿＿ | a33 |
| 34 | 最近一次住院共几天？ ＿＿＿＿天 | a34 |
| 35 | 最近一次住院共自己支付了（自费部分）多少钱？ ＿＿＿＿元 | a35 |
| 36 | 最近一次住院费用，是否可以得到下列哪类报销？（可多选）①社会基本医疗保险 ②深圳劳务工医疗保险 ③新型农村合作医疗③  商业保险 ⑤统筹医疗保险 ⑥少儿医疗保险 ⑦自费⑧其他＿＿＿＿＿＿＿＿＿ | a36 |
|  |  |  |
|  |  |  |
|  |  |  |
|  |  |  |
|  |  |  |
|  |  |  |
|  |  |  |
|  |  |  |

| 表3. 18-59岁成年人调查表 | | code |
| --- | --- | --- |
| 1 | 您是否吸烟？ ①吸烟②已戒烟 （跳至4题）③从不吸烟 （跳至6题） | b1 |
| 2 | 您当前的吸烟频度有多大？ ①经常吸（一周内≥5天） ②偶尔吸（朋友聚会或应酬时吸） （跳至4题） | b2 |
| 3 | 最近一周您一共吸了多少支烟（支）？＿＿＿＿支 | b3 |
| 4 | 您第一次吸烟是多少岁？＿＿＿＿岁 | b4 |
| 5 | （吸烟者跳答此题）如您已戒烟，已戒烟多少年？ ＿＿＿＿年 | b5 |
| 6 | 通常情况下，1周内您在密闭环境里接触二手烟的天数是？①几乎没有 ②有，具体＿＿＿＿天？ | b6 |
|  |  |  |
| 7 | 您是否饮酒？ ①目前饮酒 ②已戒酒（跳至9题） ③从不饮酒（跳至11题） | b7 |
| 8 | 下列何种描述最能说明您目前的饮酒状况？①  每周至少饮酒1-2次 ②每月饮酒1-2次 ③偶尔少量饮 | b8 |
| 9 | 您从多少岁开始饮酒？＿＿＿＿岁 | b9 |
| 10 | （目前饮酒者跳答此题）若您已戒酒，已戒酒多少年？＿＿＿＿年 | b10 |
| 11 | 近1月，你每天静坐（包括学习、工作、业余时间看电视、电脑等）的累积时间为？①<2小时 ② 2-4小时 ③ 4-8小时 ④8-12小时 ⑤>12小时 | b11 |
| 12 | 您最近半年，您参过过哪些体育锻炼？（可多选，选②-⑨的跳至14题）①几乎没有参加锻炼或健身活动②器械运动 ③健身操、舞蹈等 ④游泳 ⑤徒步走、慢跑、爬山 ⑥球类运动 ⑦体育比赛 ⑧武术、瑜伽等 ⑨其他＿＿＿＿＿＿ | b12 |
|  |  |  |
|  |  |  |
|  |  |  |
|  |  |  |
|  |  |  |
|  |  |  |
|  |  |  |
|  |  |  |
|  |  |  |
| 13 | 若您不能保证每周参加体育锻炼，原因是什么？（可多选）①从事体力活动，不需要额外运动 ②没时间锻炼 ③没有适合场所或不方便 ④身体好，不需要锻炼 ⑤不愿意活动 ⑥身体不好，不能参加锻炼 ⑦其它＿＿＿＿＿＿＿＿ | b13 |
|  |  |  |
|  |  |  |
|  |  |  |
|  |  |  |
|  |  |  |
|  |  |  |
|  |  |  |
| 14 | 最近半年，您平均每周体育锻炼几次？①  6次以上 ②3-5次 ③1-2次 ④不到1次 | b14 |
| 15 | 平均每次锻炼多少分钟（分钟）？＿＿＿＿分钟 | b15 |
| 16 | 您认为您家吃饭菜的口味如何？（可多选）①偏咸 ②适中 ③偏淡 ④偏甜 ⑤偏油 | b16 |
|  |  |  |
|  |  |  |
|  |  |  |
|  |  |  |
| 17 | 最近一周，您有几天吃早餐？① 小于1天 ②1-2天 ③3-4天 ④5-6天 ⑤每天都吃 | b17 |
| 18 | 最近一周，您吃了几天粗粮和薯类？（包括黑米、燕麦、荞麦、小米、红小豆、芸豆、莲子、薏米等）① 小于1天 ②1-2天 ③3-4天 ④5-6天 ⑤每天都吃 | b18 |
| 19 | 最近一周，您通常一天有几餐会吃蔬菜？① <1餐/天 ②1餐/天 ③2餐/天 ④3餐/天 | b19 |
| 20 | 最近一周，您有几天吃了水果？①小于1天 ②1-2天 ③3-4天 ④5-6天 ⑤每天都吃 | b20 |
| 21 | 最近一周，您有几天吃了水产品（鱼、虾、贝、蟹）？①小于1天 ②1-2天 ③3-4天 ④5-6天 ⑤每天都吃 | b21 |
| 22 | 最近一周，您进食几次奶或奶制品（酸奶、奶酪、奶片）？____次 | b22 |
| 23 | 请参考图片说说您每次进食奶或奶制品的量为多少？____毫升 ____克 | b23 |
|  |  |  |
| 24 | 平常口渴时，您通常选择什么作饮料？（单选）①  白开水②  茶/咖啡③  含糖碳酸饮料（比如可乐、汽水等）④  风味饮料（比如果味、茶味、咖啡味、乳味饮料等）⑤  牛奶、酸奶、羊奶等⑥  鲜榨果汁⑦其他____ | b24 |
| 25 | 最近一周，您有几天喝过含糖碳酸饮料（指市面上常见的瓶装、听装等含糖碳酸饮料，如可乐、水果味碳酸饮料等，但不包括苏打水、啤酒、无糖汽水等其他不含糖或低糖的碳酸饮料。）？①小于1天 ②1-2天 ③3-4天 ④5-6天 ⑤每天都喝 | b25 |
| 26 | 最近一周，您有几天喝过果汁/果味饮料（任何使用果蔬汁和糖、人工添加剂、人工香料调制的果味、蔬菜味饮料，不包括现制的鲜榨果汁。）？①小于1天 ②1-2天 ③3-4天 ④5-6天 ⑤每天都喝 | b26 |
| 27 | 您的膳食结构为：①荤素均衡 ②荤食为主 ③素食为主 | b27 |
| 28 | 您是否听说过《中国居民膳食指南》？ ①是 ②否（跳至30） | b28 |
| 29 | 平衡膳食的主要特征是： ①食物多样、谷类为主 ②荤素搭配、谷类为主 ③荤素搭配、肉类为主 ④食物多样、肉类为主 ⑤不知道 | b29 |
| 30 | 您最近一次测量体重的时间是？ ①1个月内 ②3个月内 ③6个月内④12个月内⑤一年前 ⑥记不清 | b30 |
| 31 | 最近半年，您有控制体重吗？①有 ②没有（跳转33题） | b31 |
| 32 | 您控制或减轻体重方法有哪些？（可多选）①控制饮食 ②锻炼 ③药物 ④其他 | b32 |
|  |  |  |
|  |  |  |
|  |  |  |
| 33 | 平时您主要从哪里获得健康保健知识？（可多选） ①  学校或单位②电视、广播 ③宣传栏 ④医生 ⑤同事、朋友聊天 ⑥网站 ⑦微信、app⑧书籍、报纸⑨卖保健品的人介绍 ⑩ 其它＿＿＿＿ ⑪不关注健康保健知识 | b33 |
|  |  |  |
|  |  |  |
|  |  |  |
|  |  |  |
|  |  |  |
|  |  |  |
|  |  |  |
|  |  |  |
|  |  |  |
|  |  |  |
| 34 | 您认为健康是？①身体健康 ②心理健康 ③身体+心理健康 ④身体健康+心理健康+社会适应良好 ⑤不知道 | b34 |
| 35 | 您认为正常腋下体温是多少？①  34-35℃ ②36-37℃ ③38-39℃ ④不知道 | b35 |
| 36 | 您觉得多吃盐会影响健康吗？① 会 ②不会 ③ 不清楚 | b36 |
| 37 | 您最近一次测血压是在什么时间? ①1个月内 ②3个月内 ③6个月内④12个月内⑤一年前 ⑥记不清 | b37 |
| 38 | 您认为，以下哪一个是成年人确诊高血压的诊断标准？①≥140/90mmHg ②≥120/80mmHg ③≥165/95mmHg④≥180/100mmHg ⑤ 不清楚 ⑥ 其他：＿＿＿＿＿＿＿＿ | b38 |
| 39 | 您知道确诊高血压病病人应怎样进行药物治疗吗？①  终身坚持服药 ②血压高时服药 ③有症状时服药 ④不知道 | b39 |
| 40 | 您认为，高血压会引起下列哪些并发症?（可多选）①  中风 ②心肌梗塞 ③肾病 ④视网膜病变 ⑤不清楚 ⑥其他＿＿＿＿ | b40 |
|  |  |  |
|  |  |  |
|  |  |  |
|  |  |  |
|  |  |  |
|  |  |  |
| 41 | 您认为，高血压与哪些危险因素有关：（可多选）①遗传 ②肥胖 ③高盐饮食 ④过量饮酒 ⑤吸烟 ⑥精神紧张 ⑦缺乏锻炼⑧微量元素缺乏 | b41 |
|  |  |  |
|  |  |  |
|  |  |  |
|  |  |  |
|  |  |  |
|  |  |  |
|  |  |  |
|  |  |  |
| 42 | 您认为下列哪个空腹血糖值（FBG）范围属于糖尿病高危人群？①5.1≤FBG≤6.0mmol/L ②6.1≤FBG≤7.0mmol/L③7.0≤FBG≤8.0mmol/L ④不知道 | b42 |
| 43 | 您认为，确诊糖尿病病人应怎样进行药物治疗吗？①终身坚持服药 ②血糖高时服药 ③有症状时服药 ④不知道 |  |
| 44 | 您认为，孕妇吸烟或吸二手烟会影响胎儿的正常发育吗？①  无影响 ②有影响 ③不知道 | b44 |
| 45 | 您认为，艾滋病主要通过哪些途径传播吗？（可多选） ①  性传播 ②血液传播 ③母婴传播 ④生活接触 ⑤空气传播 ⑥不知道 | b45 |
|  |  |  |
|  |  |  |
|  |  |  |
|  |  |  |
|  |  |  |
| 46 | 您认为，乙肝主要通过哪些途径传播吗？ ①  与病人或感染者一起工作、吃饭、游泳 ②可以通过性行为、输血、母婴传播 ③同病人或感染者说话、握手、拥抱 ④不知道 | b46 |
| 47 | 您认为慢性病人应采取哪些措施？（可多选） ①及时就诊 ②规范治疗 ③合理用药 ④预防并发症 ⑤提高生活质量 ⑥不知道 | b47 |
|  |  |  |
|  |  |  |
|  |  |  |
|  |  |  |
|  |  |  |
| 48 | 您认为健康生活方式包括哪些内容？（可多选） ①平衡饮食 ②保持心情平衡 ③戒烟、限酒 ④适量运动 ⑤保持充足的睡眠 ⑥讲究个人卫生 ⑦不知道 | b48 |
|  |  |  |
|  |  |  |
|  |  |  |
|  |  |  |
|  |  |  |
|  |  |  |
| 49 | 您认为如何进食蔬菜对健康有益？（深色蔬菜：深绿色、红色、桔红色、紫红色蔬菜）①每天进食蔬菜300-500克，其中浅色蔬菜必须占1/2 ②每天进食蔬菜300-500克，其中深色蔬菜必须占1/2 ③每天进食蔬菜100-300克，其中浅色蔬菜必须占1/2 ④每天进食蔬菜100-300克，其中深色蔬菜必须占1/2 ⑤不知道 | b49 |
| 50 | 你认为，成年人每日需要喝多少奶？ ①150克 ②300克 ③500克 ④不需要每天喝奶 | b50 |
| 51 | 关于鱼、禽、肉、蛋以下说法哪个是错误的？（可多选） ① 优先选择鱼肉和禽肉 ②每天吃鸡蛋，不吃蛋黄 ③不需特别限制烟熏和腌制品的进食量 ④吃畜肉应当选吃瘦肉 | b51 |
|  |  |  |
|  |  |  |
|  |  |  |
|  |  |  |
| 52 | 您觉得每天喝多少水有益健康？①3～4杯（600～800毫升） ②5～6杯（1000～1200毫升） ③7～8杯（1500～1700毫升） ④不知道 | b52 |
| 53 | 成人每天食用盐最好不超过多少克？ ①3克 ②6克 ③10克 ④不知道 | b53 |
| 54 | 成人每天食用油多少克比较好？①10～20克 ②25～30克 ③40～50克 ④不知道 | b54 |
| 55 | 以下哪种脂肪对身体最有害？①  多不饱和脂肪酸 ②单不饱和脂肪酸 ③反式脂肪酸 ④不知道 | b55 |
| 56 | 您平时购买食品是否有查看食品包装上看食品包装上的营养标签的习惯 ？①  从未看过（跳转至58题） ②很少看 ③经常看 ④每次都会看 | b56 |
| 57 | 查看食品包装上的营养标签时，应该着重注意哪些项目？（可多选）①蛋白质 ②糖 ③反式脂肪酸 ④钠 | b57 |
|  |  |  |
|  |  |  |
|  |  |  |
| 58 | 您知道中国营养学会推荐平均每天应摄入＿＿＿＿种以上食物,每周摄入＿＿＿＿种以上?①12,25 ②5,10 ③ 不知道 | b58 |
| 59 | 您知道成人体质指数（BMI）正常范围吗？ ①18.5≤BMI＜24.0 ②19.5≤BMI＜25.0 ③不知道 | b59 |
| 60 | 您知道成人体质指数（BMI）的计算公式吗？ ①BMI=身高（m）^2^/ 体重(kg) ②BMI=体重(kg)/身高(m)^2^ ③不知道 | b60 |
| 61 | 您平时就诊最多的医疗机构是?（单选）①  私人诊所 ②社区卫生服务中心（社康中心） ③区级综合医院 ④市级综合医院 ⑤区级或市级专科医院 ⑥省级以上医院 ⑦其他＿＿＿ | b61 |
| 62 | 选择上述单位就诊的主要原因是什么?（可多选） ①距离近 ②收费合理 ③技术水平高 ④设备条件好 ⑤药品丰富 ⑥服务态度好 ⑦定点单位⑧有熟人　　 ⑨有信赖医生 ⑩其它＿＿＿＿ | b62 |
|  |  |  |
|  |  |  |
|  |  |  |
|  |  |  |
|  |  |  |
|  |  |  |
|  |  |  |
|  |  |  |
|  |  |  |
|  |  |  |
| 63 | 您家到最近的社区卫生服务中心（社康中心）通常需要多少时间？①＿＿＿（分钟）②没去过，不知道在哪里（跳转80题） | b63 |
| 64 | 您到那里去的目的？（可多选） ①看病 ②开药 ③慢性病随访 ④咨询 ⑤针灸、理疗 ⑥预防接种 ⑦儿童查体 ⑧其他＿＿ | b64 |
|  |  |  |
|  |  |  |
|  |  |  |
|  |  |  |
|  |  |  |
|  |  |  |
|  |  |  |
|  |  |  |
| 65 | 社区卫生服务中心（社康中心）的医生会不会和您交谈本区需注意的一些健康问题（如流感）或近期居民患病特点吗？ ①是 ②否 | b65 |
| 66 | 你听说过“家庭医生签约服务”吗？①听说过 ②没有（跳至73题） | b66 |
| 67 | 您是通过哪些渠道了解“家庭医生签约服务”的？（可多选）①  社区卫生服务人员 ②广播电视 ③报刊 ④网络 ⑤其他 ⑥不知道 | b67 |
|  |  |  |
|  |  |  |
|  |  |  |
|  |  |  |
|  |  |  |
| 68 | 您或家里人是否有签约家庭医生？ ①是 ②否(跳至73题) ③不知道(跳至73题) | b68 |
| 69 | 您认为开展家庭医生签约服务，对家庭成员疾病防治的作用？①  非常有用 ②比较有用 ③一般 ④作用较小 ⑤根本没用 | b69 |
| 70 | 签约家庭医生后，您是否会首选到与签约的家庭医生处进行诊疗？ ①是 ②否 | b70 |
| 71 | 您是否认为社区需要为签约对象提供预约门诊服务？ ①是 ②否 | b71 |
| 72 | 您最希望通过哪些方式向家庭医生进行互动的健康咨询？（可多选） ①亲身前往医疗机构 ②电话咨询 ③电子邮件 ④社区服务专用软件 ⑤短信⑥即时通讯服务（如QQ\微信） | b72 |
|  |  |  |
|  |  |  |
|  |  |  |
|  |  |  |
|  |  |  |
| 73 | 过去一年内，您是否参加过社区卫生服务中心（社康中心）的健康讲座？ ①是 ②否 | b73 |
| 74 | 过去一年内，您是否看过社区卫生服务中心（社康中心）的宣传资料？ ①是 ②否 | b74 |
| 75 | 您知道社区卫生服务中心（社康中心）会提供慢性病管理服务吗？ ①是 ②否 | b75 |
| 76 | 您认为社区卫生服务中心（社康中心）看病环境整洁舒适吗？ ①非常满意 ②满意 ③一般④不满意 ⑤非常不满意 ⑥没去过，无法评价 | b76 |
| 77 | 您对社区卫生服务中心（社康中心）医护人员工作态度满意吗？①  非常满意 ②满意 ③一般④不满意 ⑤非常不满意 ⑥没去过，无法评价 | b77 |
| 78 | 医护人员给与您充分的时间进行解释和交流是否满意？ ①  非常满意 ②满意 ③一般 ④不满意 ⑤非常不满意 ⑥没去过，无法评价 | b78 |
| 79 | 您认为社区卫生服务中心（社康中心）医疗技术水平？ ①  非常满意 ②满意 ③一般 ④不满意 ⑤非常不满意 ⑥没去过，无法评价 | b79 |
| 80 | 在过去的30天中，您感到过紧张、焦虑或烦躁吗？ ①经常有 ②有时有 ③偶尔有 ④没有 | b80 |
| 81 | 在过去的30天中，您感到过绝望、沮丧，什么都没劲，以至于没什么事情能让您开心吗？ ①经常有 ②有时有 ③偶尔有 ④没有 | b81 |
| 82 | 在过去的30天中，您感到自己一无是处、做什么事情都费劲吗？①经常有 ②有时有 ③偶尔有 ④没有 | b82 |
| 83 | 如果您想外出旅行一天（如：下郊游或去山里），是否能够很容易找到人和您同行。①经常有 ②有时有 ③偶尔有 ④没有 | b83 |
| 84 | 你会经常找到朋友或亲戚倾诉你的烦恼和恐惧吗？①经常有 ②有时有 ③偶尔有 ④没有 | b84 |
| 85 | 如果您生病了，是否能够容易找到人帮忙处理日常家务。①经常有 ②有时有 ③偶尔有 ④没有 | b85 |
| 86 | 如果您某天下午决定当晚去看电影，是否能够容易找到人相伴。①经常有 ②有时有 ③偶尔有 ④没有 | b86 |
| 87 | 您会常被别人邀请去参加各种活动（比如逛街，吃饭，看电影，或做其它非工作上的事情）。①经常有 ②有时有 ③偶尔有 ④没有 | b87 |
| 88 | 如果您想找人共进午餐，是否很容易就找人相伴。①经常有 ②有时有 ③偶尔有 ④没有 | b88 |
| 89 | 如果您遇到困难、一筹莫展时，是否能够打电话找到人前来帮忙。①经常有 ②有时有 ③偶尔有 ④没有 | b89 |
| 90 | 您认为居民是否需要学习急救知识？ ①非常应该 ②应该 ③不重要 ④不知道是否应该学习 | b90 |
| 91 | 发生意外事故后，您的第一反应是？ ①紧张害怕，不知所措 ②拨打急救电话并等待救援 ③拨打急救电话，并在救护车来之前施以急救措施 ④立即施以救援，时间就是生命 | b91 |
| 92 | 您听说过心肺复苏吗？①知道 ②不知道（跳至95题） | b92 |
| 93 | 心肺复苏术是抢救生命最重要的技术，您知道它的第一步是什么吗？① 人工呼吸 ②开放气道 ③胸外心脏按压 | b93 |
| 94 | 您觉得心跳骤停几分钟后为急救的黄金阶段？① 2-4 ②4-6 ③6-8 ④8-10 | b94 |
| 95 | 您认为，以下哪种是中暑的急救方法 （可多选）① 喝凉开水或淡盐水 ②喝温热水，以便增强血液循环 ③掐人中、合谷穴 ④实施心肺复苏术 | b95 |
|  |  |  |
|  |  |  |
|  |  |  |
| 96 | 您认为，对于烫伤的处理，哪项是正确的？①立即用凉水冲洗10-15分钟 ②在伤口上涂牙膏、酱油等 ③用无菌纱布或棉布紧紧捆扎 | b96 |
| 97 | 您生活或工作的地方是否设置灭火器和消防栓？ ①有 ②没有 | b97 |
| 98 | 您知道灭火器和消防栓正确使用方法吗？ ①知道 ②不知道 | b98 |
